# A Survey on Use of Rapid Tests and Tuberculosis Diagnostic Practices by Primary Health Care Providers in South Africa: Implications for the Development of New Point-of-Care Tests

**DOI:** 10.1371/journal.pone.0141453

**Published:** 2015-10-28

**Authors:** Malika Davids, Keertan Dheda, Nitika Pant Pai, Dolphina Cogill, Madhukar Pai, Nora Engel

**Affiliations:** 1 Lung Infection and Immunity Unit, Division of pulmonology and UCT lung Institute, Department of Medicine, University of Cape Town, Anzio Road, Cape Town, South Africa; 2 Division of Clinical Epidemiology, Department of Medicine, McGill University and McGill University Health Centre, V Building, Royal Victoria Hospital, 687 Pine Avenue West, Montreal, H3A1A1, Canada; 3 McGill International TB Centre, Department of Epidemiology & Biostatistics, McGill University, 1020 Pine Ave West, Montreal, QC H3A 1A2, Canada; 4 Department of Health, Ethics & Society, Research School for Public Health and Primary Care, Maastricht University, Postbus 616, NL - 6200 MD, Maastricht, The Netherlands; California Department of Public Health, UNITED STATES

## Abstract

**Background:**

Effective infectious disease control requires early diagnosis and treatment initiation. Point-of-care testing offers rapid turn-around-times, facilitating same day clinical management decisions. To maximize the benefits of such POC testing programs, we need to understand how rapid tests are used in everyday clinical practice.

**Methods:**

In this cross-sectional survey study, 400 primary healthcare providers in two cities in South Africa were interviewed on their use of rapid tests in general, and tuberculosis diagnostic practices, between September 2012 and June 2013. Public healthcare facilities were selected using probability-sampling techniques and private healthcare providers were randomly selected from the Health Professional Council of South Africa list. To ascertain differences between the two healthcare sectors 2-sample z-tests were used to compare sample proportions.

**Results:**

The numbers of providers interviewed were equally distributed between the public (n = 200) and private sector (n = 200). The most frequently reported tests in the private sector include blood pressure (99.5%), glucose finger prick (89.5%) and urine dipstick (38.5%); and in the public sector were pregnancy (100%), urine dipstick (100%), blood pressure (100%), glucose finger prick (99%) and HIV rapid test (98%). The majority of TB testing occurs in the public sector, where significantly more providers prefer Xpert MTB/RIF assay, the designated clinical TB diagnostic tool by the national TB program, as compared to the private sector (87% versus 71%, p-value >0.0001). Challenges with regard to TB diagnosis included the long laboratory turn-around-time, difficulty in obtaining sputum samples and lost results. All providers indicated that a new POC test for TB should be rapid and cheap, have good sensitivity and specificity, ease of sample acquisition, detect drug-resistance and work in HIV-infected persons.

**Conclusion/significance:**

The existing centralized laboratory services, poor quality assurance, and lack of staff capacity deter the use of more rapid tests at POC. Further research into the practices and choices of these providers is necessary to aid the development of new POC tests.

## Introduction

Point-of-care testing (POCT) has gained much attention in recent years, especially in resource- limited settings, where the need for rapid, cost-effective diagnosis is paramount. The use of tests that are rapid, accurate, and deployable at POC are projected to substantially reduce tuberculosis (TB)-related morbidity and mortality [1] and several new diagnostic tests are in the pipeline for TB [2].

TB is the major cause of morbidity and mortality in South Africa [3]. While South Africa’s diagnostic eco-system is characterized by centralized testing and thus the majority of TB testing happens in the reference or centralized laboratories [4], several studies have assessed the feasibility of implementing Xpert MTB/RIF assay (an automated diagnostic test, which rapidly identifies *Mycobacterium tuberculosis* and Rifampicin resistance), sputum smear microscopy and/or urine-LAM tests (a urine dipstick test that diagnosis TB using lipoarabinomannan antigen detection) at the point of care [reviewed in [1]]. Particularly debated is the utility of Xpert MTB/RIF assay as a POC test in clinic settings [5,6].

The definition of POC testing is in evolution [7–10], yet critical elements of POCT remain: a) rapid turn-around-times (TATs) to allow for quick diagnosis and b) referral or treatment decisions completed within the same patient encounter (i.e. POC continuum) or, at the very minimum, with results delivered on the same day.

Currently, over 100 rapid tests are available on the market for a variety of diseases such as diabetes, influenza, anaemia, malaria, syphilis, hepatitis and HIV [11]. However, the majority of studies assessing existing rapid tests focus on accuracy, cost-effectiveness and clinical outcomes; and are single disease focused [12–14]. It is now apparent that test accuracy does not automatically translate into clinical impact [7]. Consequently, a greater understanding of the overall diagnostic ecosystem is needed for successful development and implementation of new tests.

We have shown elsewhere that rather than the test processing time, the entire turn-around time from sample acquisition to patient treatment initiation matters for diagnostic delay [4]. Moreover, professionals adapt their diagnostic practices to overcome challenges, often further compounding diagnostic delay [4]. Providers might choose tests that are inaccurate yet economically more rewarding [15]. A significant proportion of patients in high burden settings fail to return to collect their sputum smear results [16]. These examples highlight the need for a better understanding of routine diagnostic practices. Understanding the practices and perspectives of healthcare providers, who utilize tests on a daily basis, will help to develop better tests and testing programs that work at POC and will offer important lessons for manufacturers, funders, policymakers and public health officials.

To address this knowledge gap, we examined what rapid tests are conducted at the primary care level, in the public and private sector, in South Africa and why healthcare workers choose these rapid tests. To that effect, we surveyed primary healthcare providers with regard to their use of rapid tests. In the context of South Africa, we focused more closely on TB and examined how primary healthcare providers diagnose TB in their specific setting and what their perspectives are on an ideal POC TB test.

## Methods

We conducted a cross-sectional survey of 400 primary healthcare providers (doctors and nurses) during September 2012 and June 2013. The study was conducted in two major urban cities in South Africa (Cape Town and Durban), from both the private sector (n = 198 doctors and n = 2 nurses) and public sector (n = 200, 57 doctors and 143 nurses). South Africa has a two-tier healthcare system consisting of public and private healthcare providers. Primary healthcare centres offer basic services to treat and diagnose patients’ symptoms and manage acute clinical injuries, secondary healthcare centres offer specialized- outpatient treatment and tertiary healthcare centres offer specialized inpatient treatment [17]. In the public sector, nurses provide most clinical services while doctors are only consulted in complicated cases or visit clinics weekly to run specialized clinics, such as Anti-Retro Viral (ARV) clinics for HIV therapy [17]. Currently, all laboratory diagnostic tests are conducted through the National Health Laboratory Service (NHLS). Testing is thus largely centralized. In exceptional cases, some primary health care clinics have mobile laboratories to conduct basic TB diagnostic tests (e.g. Sputum smear, Xpert MTB/RIF assays) [4]. In contrast, the private sector comprises of mostly doctors catering to middle- and high-income earners who tend to be covered by medical insurance schemes. The study sites (Durban and Cape Town), similar to the rest of South Africa, suffer from concurrent epidemics of HIV and TB. While both Cape Town and Durban have a similar TB incidence (1076 *vs*. 1033 per 100,000, respectively) [18], the proportion of HIV infected individuals is much higher in Durban (16.9%) compared to Cape Town (5%) [19]. In both cities, TB and HIV are usually diagnosed at the primary healthcare level in both the private and public sectors.

For this study, primary healthcare clinics were randomly selected by systematic probability sampling from The Western Cape and KwaZulu-Natal Provincial list of public healthcare clinics respectively. There are a total of 136 primary healthcare facilities in the eThekwini municipality of Durban and 72 in Cape Town. After sampling, 12 facilities in Cape Town and 15 facilities in Durban were selected for the study. All nurses and doctors at the selected public clinics were interviewed, and none refused to participate in the study. General practitioners and family practice doctors from the private sector were randomly selected from the Health Professional Council of South Africa list. A total of 894 private doctors were approached to participate in the study. 694 of the private doctors refused to participate in the study because of their busy time schedules. A sample size of 200 was pre-specified in the protocol, and recruitment ended when the predetermined sample size was reached.

The survey was developed by trained social scientists, administered by a qualified researcher, and took approximately 30–45 minutes to complete. The questionnaire comprised of both multiple choice and open questions, where providers were asked to provide further explanation to their answers (see S1 Study Survey). Survey questions focused on the types of rapid diagnostic tests being administered in primary healthcare facilities, methods of TB diagnosis and associated diagnostic challenges and criteria for an ideal TB POC test. Questions related to the provider’s preference of TB diagnostic tests and their description of an ideal POC test, required the provider to grade their choices using a 10-point Likert scale (one being not important and ten being very important). Additionally the researcher administering the survey recorded anecdotal notes. These notes covered aspects such as the clinic department in which the doctor or nurse worked in, general discussions taking place whilst conducting the survey and set-up/infrastructure of the clinic.

All participants signed informed consent forms before the survey was conducted. Ethical approval to conduct this study was obtained from the Western Cape Provincial Department of Health, KwaZulu-Natal Provincial Department of Health, the University of Cape Town and McGill University Health Centre.

All surveys were dually entered into a Microsoft Access database by two independent capturers. The data was validated using EpiInfo. Qualitative questions were analysed using Nvivo (QSR International, version 9.2.1). A social scientist and microbiologist developed a coding scheme jointly. The data was grouped into emerging themes. Graphpad Prism (Graphpad USA, version 6; Mann-Whitney t-test), and EpiTools (http://epitools.ausvet.com.au/) 2-sample z-test was used to compare sample proportions.

## Results

### Characteristics of participants

Of the 400 providers interviewed, 50% were from Durban and 50% from Cape Town. The majority of the surveyed providers in the private sector were medical doctors (98%, Table 1) as compared to a majority of nurses in the public sector (72%, Table 1). 56.5% of the public providers had been practicing as medical professionals for more than 5 years, compared to 83% in private sector (p-value < 0.001). Providers working in the public sector saw more patients per day than those in the private sector. 77% of public doctors and nurse reported seeing 21–30 patients per day, while 60.5% of private providers saw 11–20 patients per day. Furthermore, public providers mainly saw patients with low monthly income (79.5%, Table 1), whereas private providers mainly had clients with middle-high monthly income (45%, Table 1). 95% of public providers did not charge their patients for consultations, as the government runs the clinics. 94.5% of providers from the private sector typically charged medical aid rates for consultations, indicating that most of their patients had medical insurance (Table 1).

**Table 1:** Demographic details of providers and clinical practice.

Type of provider	Providers	
	Public sector	Private sector
Doctor n (%)	57 (28.5)	98 (99)
Nurse n (%)	143 (71.5)	2 (1)
**Median years practicing as medical professional:**		
Less than 6 months	0.5	0
More than 6 months but less than a year	3	1
More than 1 year but less than 5 years	40	16
More than 5 years	56.5	83
**Monthly household income of patients:**		
Low income	79.5	2
Low-middle income	19	9,5
Middle income	1	38
Middle-high income	0	45
High income	0.5	5,5
**Number of daily consultations (% providers):**		
0–2	0	0
3–10	0.5	26.5
11–20	6	60.5
21–30	77	11.5
31–50	14	1.5
51–100	2.5	0
101–200	0	0
**Number of monthly TB patients (% providers):**		
1–2	9.5	6
3–10	15	5.5
11–20	12	1
21–30	4.5	0
31–50	6.5	0
51–100	17.5	1
101–200	30	1
More than 200	3.5	0
Other: zero	0	38
Other: rarely/ less than 10 per year	0	47.5
**Most common infectious disease diagnosed:**		
Diarrheal disease	46	65.5
Respiratory tract infection	54.5	99
Sexually transmitted infections	11	5
HIV/AIDS	46	18.5
Tuberculosis	59	7
Hepatitis	2.5	5
**Typical fee for an initial consultation (median)**		
Medical aid	1.5	94.5
No charge	95	2
Does not want to reveal	3.5	0

### Rapid tests performed by providers to make quick management decisions

The survey asked about the use of specific rapid tests (e.g. pregnancy, glucose, HIV, malaria, Xpert MTB-RIF, syphilis, hepatitis, influenza, dengue, typhoid, streptococcal pharyngitis, blood pressure). In addition, providers were able to indicate other rapid tests not listed on the survey.

In the private sector, the most common rapid tests performed by primary healthcare doctors or nurses were the blood pressure (99.5%) and glucose finger prick test (89.5%), and to a lesser extent urine dipstick (38.5%), cholesterol (23%), HIV rapid test (21%), haemoglobin (19.5%) and pregnancy test (15.5%). In the public sector clinics, the most commonly performed rapid tests were the pregnancy test (100%), blood pressure (100%), urine dipstick (100%), glucose finger-pick test (99%), and HIV rapid test (98%); and to a lesser extent haemoglobin (39.5%), syphilis (34.5%), hepatitis (14%) and cholesterol (3.5%; Table 2).

**Table 2:** Overview of rapid tests offered by primary healthcare providers.

Rapid test	Number of providers conducting rapid tests (%)		Median number of rapid tests conducted per month		Median time to get the test results. (Minutes)		Who performs the rapid tests						Number of providers that make treatment decisions on the based of the rapid test results (%)		Cost to patient per rapid test. (USD)		Percentage of providers that provide post-test counselling (%)	
							Private Sector			Public Sector								
	Private sector	Public sector	Private sector	Public *	Private sector	Public sector	Provider	Support staff	Attached lab	Provider	Support staff	Lab	Private sector	Public sector	Private sector	Public sector	Private sector	Public sector
**Pregnancy**	31 (15.5)	200 (100)	12	672	15	15	20/ 31 (65)	11/ 31 (35)	0	105/ 200 (53)	95/ 200 (47)	0			7	0	8/31 (26)	114/200 (57)
**Glucose**	179 (89.5)	198 (99)	119	4423	2.7	2.7	164/179 (92)	13/ 179 (7.3)	0	104/ 198 (53)	94/ 198 (47)	0	179/ 179 (100)	198/ 198 (100)	0	0	63/ 179 (35)	79/ 200 (40)
**HIV**	42 (21.0)	196 (98)	30	782	16.8	20	30/ 42 (71)	12/ 42 (29)	0	50/ 196 (26)	145/ 196 (74)	0	37/ 42 (88)	120/ 196 (61)	6	0	42/ 42 (100)	196/ 196 (100)
**Xpert MTB-RIF**	0 (0)	30 (15)	N/A	535	N/A	124	N/A	N/A	N/A	0/ 30 (0)	0/ 30 (0)	30/ 30 (100)	N/A	30/ 30 (100)	N/A	0	N/A	18/ 30 (60)
**Syphilis**	13 (6.5)	69 (34.5)	6.15	719	13.5	13	8/ 13 (62)	5/ 13 (38)	0	47/ 69 (68)	22/ 69 (32)	0	13 / 13 (100)	69/ 69 (100)	6	0	11/ 13 (85)	66/ 69 (96)
**Hepatitis**	0 (0)	28 (14)	N/A	369	N/A	13	N/A	N/A	N/A	19/ 28 (68)	9/ 28 (32)	0	N/A	28/ 28 (100)	N/A	0	N/A	20 / 28 (71)
**Other:** **Cholesterol**	46 (23)	7 (3.5)	40	421	4.87	2	38/ 46 (83)	8/46 (17)	0	3/ 7 (43)	4/ 7 (57)	0	46/ 46 (100)	7/ 7 (100)	1.22	0	14/ 46 (30)	3/ 7 (43)
**Other:** **Urine dipstick**	77 (38.5)	200 (100)	77	4642	3.4	3	65/ 77 (84)	12/ 77 (16)	0	124/ 200 (62)	76/ 200 (38)	0	77/ 77 (100)	200/ 200 (100)	0	0	28/ 77 (36)	91/ 200 (46)
**Other:** **Blood pressure**	199 (99.5)	200 (100)	188	4626	3.17	2	184/ 199 (92)	15/ 199 (8)	0	121/ 200 (60)	79/ 200 (40)	0	199/ 199 (100)	200 / 200 (100)	0	0	54/ 199 (27)	95/ 200 (48)
**Other:** **Ultrasound**	4 (2)	0 (0)	16	N/A	12.5	N/A	2/ 4 (50)	2/ 4 (50)	0	N/A	N/A	N/A	4/ 4 (100)	N/A	25.89	0	2/4 (50)	N/A
**Other:** **Haemoglobin**	39 (19.5)	79 (39.5)	70	2381	3.95	3	32/ 39 (82)	7/ 39 (18)	0	48/ 79 (60)	31/ 79 (40)	0	39 / 39 (100)	79/ 79 (100)	0	0	12/ 39 (30)	51/ 79 (65)
**Other:** **X-ray**	7 (3.5)	0 (0)	8.57	N/A	15	N/A	0/ 7 (0)	7/ 7 (100)	0	N/A	N/A	N/A	7/ 7 (100)	N/A	34	N/A	5/ 7 (71)	N/A

*Median per clinic.

The median number of tests conducted per month at each primary healthcare clinic was higher than that reported per private practice. Examples are: pregnancy (672 versus 12, respectively), glucose finger prick (4423 versus 119, respectively), HIV (782 versus 30 respectively), syphilis (719 versus 6.15, respectively), cholesterol (421 versus 40, respectively), urine dipstick (4642 versus 77, respectively), blood pressure (4626 versus 188 respectively) and haemoglobin tests (2381 versus 70, respectively) (Table 2). These tests are offered at no cost to patients in the public sector. In the private sector, some rapid tests (for example, blood pressure, glucose and urine dipstick) are included in the doctor’s consultation fee, whereas HIV, hepatitis and cholesterol rapid tests are usually offered at an additional cost to the patient (price range 6–26 USD; Table 1).

In the public sector, HIV, cholesterol and haemoglobin rapid tests are mainly (more than 50% of tests) conducted by support staff (such as triage nurses, community healthcare workers or trained counsellors and laboratory technicians; Table 2) and not by the interviewed doctor or nurse. The general clinic set-up in South Africa might explain this result. Many clinics have a triage station where dedicated nurses based at this station conduct a range of rapid tests. If a doctor/nurse requires a rapid test or a sample for laboratory-based testing, they refer the patient to the triage station. Consequently, nurses based at the triage station conduct more rapid tests than other clinic nurses (derived from anecdotal notes).

The majority of all 400 doctors and nurses (61–100%) indicated that the rapid test results impact their treatment or patient management decision (Table 2). There were no significant differences between public and private providers’ median turn-around time of performing rapid tests such as pregnancy (15 min. versus 15 min.), glucose (2.7 min. versus 2.7 min.), HIV (16.8 min. versus 20 min.) and syphilis (13.5 min. versus 13 min.).

In response to questions about the main reason for conducting these rapid tests, 85% of the providers from the private sector indicated that they conduct rapid tests because the results are accurate, 80% because the results are available immediately, and 54% because the tests are easily available in their setting. Similarly in the public sector, 79% conducted rapid tests because they are accurate, 76% because results are available immediately, and 61% because the tests are recommended by the national guidelines.

However, the doctors from the private sector indicated that the main reasons for not conducting additional rapid tests is the existence of a well set-up, centralized laboratory system, the lack of quality assurance of rapid tests, and a lack of capacity to conduct many types of rapid tests in routine clinic settings. Public sector doctors and nurses indicated that the most common reason for not using rapid tests is not being recommended by the guidelines and the existence of a centralized laboratory system.

### TB testing at primary healthcare facilities

59% of the 200 public providers and 53% of the 200 private providers reported investigating at least one case of suspected TB in their career. Yet, the majority of TB testing in South Africa happens in the public sector. Each public clinic sends significantly more biological samples to the laboratory for TB testing per month, compared to private practitioners (139 samples versus 1.79 per month, p-value <0.001; Table 3).

**Table 3:** Characteristics of TB diagnosis in the public and private healthcare sectors.

	Pulmonary TB	MDR-TB	XDR-TB	Extra-pulmonary TB
**Number of providers reporting diagnosis of TB n (%)**				
Private sector	112/200 (56)	31/200 (15.5)	2/200 (1)	9/ 200 (4.5)
Public sector	119/200 (59.5)	70/ 200 (35)	8/ 200 (4)	34 / 200 (17)
p- value (private vs. public)	0.417	<0.0001	0.054	<0.0001
**Median number of biological samples sent to lab for TB testing per month**				
Private	1.79	1.16	1	1.55
Public	139	8.84	1.5	10.89
p- value (private vs. public)	<0.001	<0.001	0.466	<0.001
**Median time from diagnosis to patient receiving lab results/ days**				
Private	2.57	3.8	30	7.27
Public	3	2.53	20.63	4.61
p- value (private vs. public)	0.897	<0.001	0.624	0.032
**Median time from TB suspect initial visit to TB treatment initiation/ days**				
Private	0.87	1.71	30	2.6
Public	2.77	2.63	23.75	3.8
p- value (private vs. public)	<0.001	<0.001	0.793	0.780
**Number of providers who start empirical TB treatment while awaiting lab results n (%)**				
Private	37/112 (33)	9/31 (29)	2/2 (100)	6/9 (66)
Public	31/ 119 (26)	22/ 70 (31)	5/ 8 (63)	28/ 34 (82)
p- value (private vs. public)	0.1248	0.6625	0	0.0003
**Challenges associated with TB diagnosis n (%)**				
No challenges	163 (41)			
No comment, I never/ rarely diagnose TB patients	152 (38)			
Obtaining a sputum sample from patients can is difficult	51 (13)			
Long laboratory turn around time	15 (4)			
Laboratory results are lost clinic and does not reach patient folder	7 (2)			
Xpert MTB RIF assay is expensive	18 (4)			

The majority of TB testing at primary healthcare facilities concerns drug sensitive TB. 15% of the 200 private providers reported testing/diagnosing MDR-TB patients and 1% reported suspecting XDR-TB and 4.5% extra-pulmonary TB cases (Table 3). In the public sector, 35% reported testing/diagnosis MDR-TB cases, 4% XDR-TB cases and 17% extra-pulmonary cases (Table 3). The low number of tests used for drug resistant TB and extra-pulmonary TB at primary health care facilities could be attributed to the limited testing capacity at these facilities. For instance, a doctor who suspects extra-pulmonary TB will refer the patient to a hospital for further testing, because the clinic does not have the capacity to perform specialized procedures such lymph nodes aspirates, biopsy of site infected and/or pleural effusion drainage (derived from anecdotal notes).

**Fig 1:**
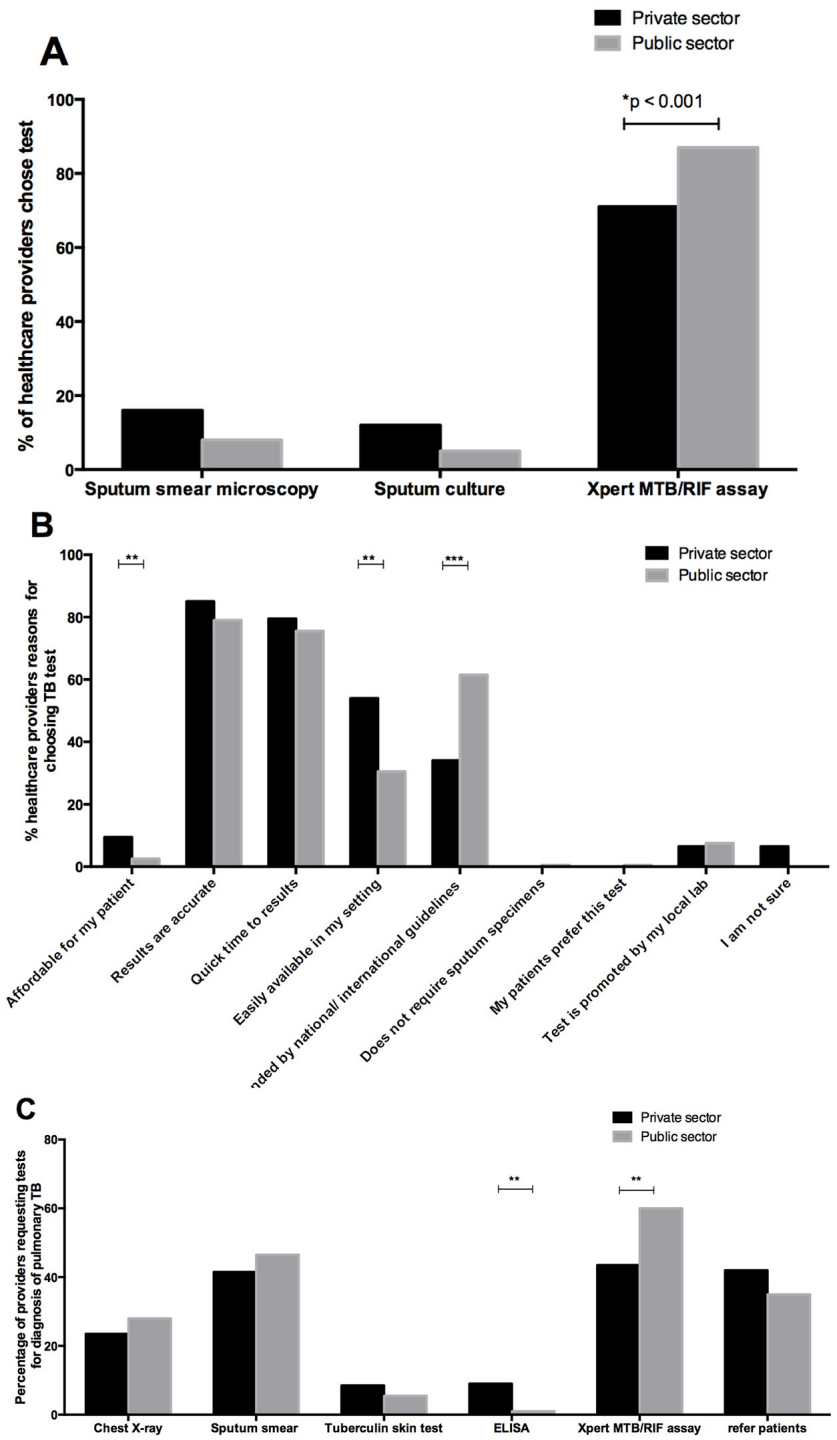
(A) Preference of TB tests available to healthcare providers, (B) justification of their preference.

When the providers were asked, which tests they order to diagnose pulmonary TB, they reported using a combination of tests, such as the Xpert MTB/RIF assay (43% private providers and 60% public providers; p-value >0.001), sputum smear microscopy (41.5% private providers and 46.5% public providers), chest radiographs (23.5% private providers and 28% public providers), tuberculin skin tests (8.5% private providers and 5.5% public providers) and blood ELISA (9% private providers and 1% public providers; p-value > 0.001) (Fig 1C). When the providers were asked to choose one preferred test to diagnose TB, significantly more providers from the public sector indicated Xpert MTB/RIF assay as compared to the private sector (87% versus 71%, p-value >0.001; Fig 1A). Based on anecdotal notes and discussions during survey administration, the reasons for this discrepancy in preferences are the lack of communication between government and private healthcare providers (who are often not aware of new diagnostic tools or what the government guidelines are); and the fact that the nurses who manage TB diagnostics in the public sector follow governmental guidelines and algorithms more strictly than private doctors. In fact, when the providers where asked to provide reasons for their answer, 76% of public doctors and nurses indicated that they choose the Xpert MTB/RIF assay because it is recommended by the national guidelines (Fig 1B).

Although 40% of all providers felt that they had no challenges associated with TB diagnosis; 13% felt that they had difficulty in obtaining a sputum sample, 4% felt that the laboratory turn-around time was too long; 2% indicated problems with results being lost at the clinic; and 4% felt that the Xpert MTB RIF assay was too expensive (Table 3).

### Criteria for an ideal POC test for TB

**Fig 2:**
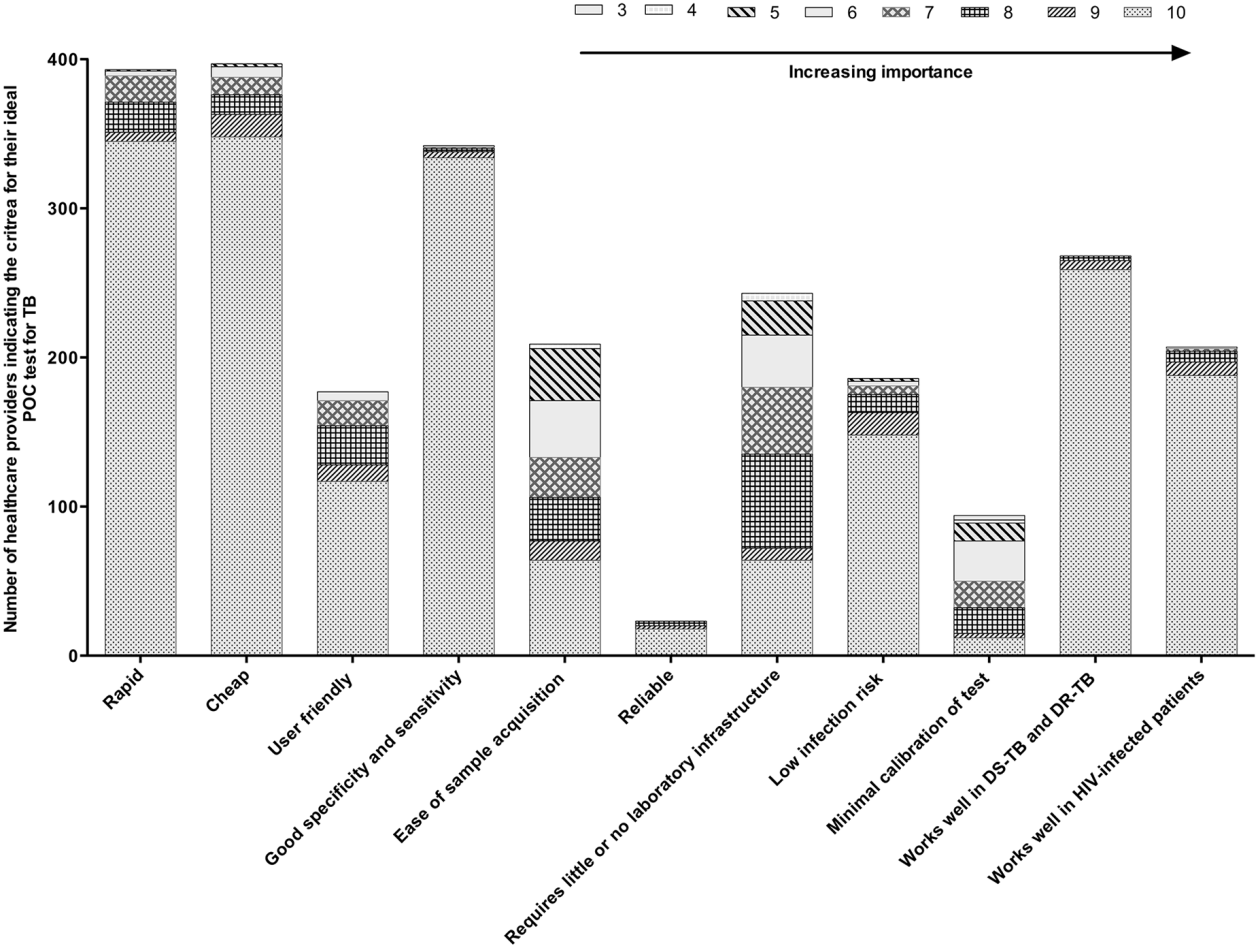
Choice of criteria for an ideal POC test for TB stratified by importance, as indicated by healthcare providers.

Out of the different ideal criteria for a new POC TB test offered in the survey (Fig 2), both public and private providers indicated that a new POC test for TB should be rapid, cheap, have good sensitivity and specificity and ease of sample acquisition. When asked to define rapid and cheap, the responses from providers varied. 23% of providers defined rapid as 10–15 minutes, 17.3% said less than 5 minutes, whilst the large majority 59% felt that a 24-hour turn-around-time would work as well (Table 4), because patients would prefer to return the next day instead of waiting hours at the clinic for results. This situation would likely be different in rural parts of South Africa, where patients would need to commute hours to the nearest clinic and thus would rather prefer to wait [4]. When asked to define what they meant by “cheap”, the majority indicated that the test should not cost more than 200 Rand (~16 USD, current exchange rate).

**Table 4:** Time requirements of a rapid POC test according to both private and public healthcare provider.

	Provider from private sector	% Provider from public sector
**>1 minute**	0.5	0
**1–5 minutes**	5	4.5
**6–9 minutes**	1	5
**10–15 minutes**	26.5	25
**16–30 minutes**	29.5	16
**1–2 hours**	18	25
**2–4 hours**	1	8.5
**4–12 hours**	0	0.5
**12–24 hours**	18	14.5
**> 24 hours**	0.5	1

In response to the open questions on ideal criteria for a new POC TB test, the surveyed doctors and nurses indicated that the test must be able to diagnose drug resistant TB, should work well in HIV-infected persons, and be user-friendly. Fig 2, describes a general overview of desired POC test criteria as described by health care providers.

## Discussion

There are no published studies on which rapid tests are conducted by primary healthcare facilities in South Africa. Most existing publications focus on the HIV rapid tests and have shown the benefits of using the HIV rapid tests in high burden countries with limited healthcare facilities [20]. Our data indicates that all primary healthcare doctors and nurses conduct one or more rapid tests in their practice, such as the pregnancy test, finger prick glucose, HIV test, etc. However, providers in the public sector perform more rapid tests than those in the private sector. Our data indicates that patients seeking care from private providers are more likely to have medical insurance and providers often request laboratory based diagnostic tests. The shorter turn-around time of rapid tests makes them more appealing to public healthcare settings, where the patient load at a clinic is significantly higher compared to a private practice (Table 2).

The majority of providers specified that they use rapid tests because it allows them to make a management decision within the same patient encounter, and because the tests are available in their setting or recommended by the guidelines. Furthermore, the centralized laboratory infrastructure in South Africa, respective policy guidelines (in the public) and ease of ordering laboratory-based tests (in the private) deter additional on-site testing with rapid tests. This highlights how healthcare practices and the particular testing infrastructure influence the use of POC tests, and points to the need to understand practices of testing and diagnosing on the ground before and during implementation of new diagnostic tools.

Although 40% of the providers indicated that they had no challenges in diagnosing TB, 13% indicated that obtaining a sputum sample is challenging. Other factors, such as long laboratory turn-around-time, lost results and high cost of Xpert MTB/RIF assay, were also mentioned as challenges by 10% of providers. These challenges reflect limitations of the available technology and the functioning of the healthcare system. Diagnostic delays due to long laboratory turn-around-times, transport issues, sample quality and lost samples/results are common in the public primary care settings in South Africa [4]. Our data suggests that in urban settings, TB diagnosis mostly occurs in public primary healthcare facilities. Similarly, Pronyk *et al* [21] found that most patients in rural areas in South Africa seek a TB diagnosis from public providers. TB is associated with low socio-economic status [22] and patients often seek medical care from facilities offering free services, despite the poor clinical service. [4]

Healthcare providers indicated that their ideal criteria for a new TB POC test are: rapid, cheap, good specificity and sensitivity, ease of sample acquisition/user friendliness, ability to detect drug-resistance and diagnose TB in HIV-infected persons. Interestingly, the definition of “cheap” from the perspective of primary health care providers equated to a median value of 16 USD. This is very comparable to the current cost of an Xpert MTB/RIF assay in the public sector (20USD, as indicated by the national health laboratory service) while the price in the private sector exceeds this (± 100USD, as indicated on the price list of Pathcare, the largest private laboratory in South Africa).

These results further reiterate the complexity of the healthcare system and the need for test developers to look past the accuracy and performance of test, but to evaluate their pragmatic benefits and the way tests are put to use in daily practice.

The experiences with the widespread rollout of the Xpert MTB/RIF assay in South Africa illustrate the challenges of implementing a new diagnostic test. The Xpert MTB/RIF assay promises to rapidly and accurately diagnose TB and to provide information on rifampicin resistance within 2 hours [23]. It thus promises to dramatically cut delays in diagnosing to effectively prevent disease transmission; a fundamental concern of TB control in high-burden settings. However, diagnostic delays continue to occur despite implementation of the Xpert MTB/RIF assay. Our data shows that although most private/public providers use the Xpert MTB/RIF assay, patients are only initiated on treatment 2–3 days later. Other studies have shown similar results [4,23], and explained these with backlogs created by the centralized testing system [4]. Additionally, its implementation as a point-of-care test within a primary healthcare facility requires the appointment of 2.5 additional nurses to ensure same-day results and follow-up [24].

Effective implementation of a new test needs to go beyond simply placing the test within the existing infrastructure of a primary care facility or laboratory [7]. For instance, the motivation and training of staff at these healthcare facilities needs to be considered. If the staff is unaware of the new technology or do not understand it, then they may be less inclined to conduct or request the test in their clinical practice [25]. The over burdened healthcare system in South Africa is another important concern. Healthcare facilities have an overwhelming patient load, which often result in long waiting times and extended diagnostic turn-around times [26]. Furthermore, patients accessing healthcare in the public sector often cannot afford to pay for diagnostic tests privately. In contrast, the private sector has a lower TB caseload, because private patients suspected of TB are often referred to the public primary healthcare clinics for diagnosis (study anecdotal notes).

Our survey had several limitations. The survey relied on self-reports by primary healthcare providers as to which tests they conduct. We did not validate the data obtained by the providers through, for instance, requesting proof of purchase of tests conducted or validating medical records. The healthcare providers could have over- or underestimated the amount of rapid tests conducted per month. Our sample of private sector doctors may have been impacted by the refusal rate.

In conclusion, this survey allows us to quantify which providers are conducting rapid tests at a primary healthcare level in South Africa, and which type of rapid tests are commonly used. Additionally, we were able to quantify differences in TB diagnostic practices in the private versus public healthcare settings. Further qualitative research is necessary to understand the choices made by providers and the differences between public and private sectors.

## Supporting Information

S1 Study Survey The study survey tool used to conduct quantitative interviews with healthcare providers. (DOCX)
