# Supplementary material for: A Survey on Use of Rapid Tests and Tuberculosis Diagnostic Practices by Primary Health Care Providers in South Africa: Implications for the Development of New Point-of-Care Tests
Source: PLoS One. 2015 Oct 28;10(10):e0141453. doi: 10.1371/journal.pone.0141453 (PMC4624929; doi:10.1371/journal.pone.0141453)
Supplement: S1 Study Survey — (DOCX) [file pone.0141453.s001.docx]

**University of Cape Town, South Africa and McGill University, Montreal**

**Quantitative survey on POC testing by primary healthcare providers in South Africa**Primary health care provider questionnaire

Study ID

Interviewer’s name: _____________________________________________________________

Primary health care provider’s name: ______________________________________________

Primary health care provider’s address

Street: __________________________________________

Town: ­­­­­­­­__________________________________________

Suburb: _________________________________________

Telephone number: _______________________________

Mobile number: __________________________________

Date of the interview: __________________

Informed consent obtained? Yes / No

**After administration of survey, store this first page separately from the rest of the questionnaire.**

**Section 1 – Primary health care provider profile**

Study ID

| Q. No | Questions and Filters | Coding Categories | | Codes |
| --- | --- | --- | --- | --- |
|  | What type of health care provider are you? | Medical doctor | | 1 |
|  |  | Nurse | | 2 |
|  |  | Other, please specify:  _______________________________ | | 3 |
|  | Please answer all questions in your capacity as a: | A doctor from the private sector | | 1 |
|  |  | A doctor from the public sector | | 2 |
|  | How long have you been practicing medicine/nursing? | Less than 6 months | | 1 |
|  |  | More than 6 months but less than a year | | 2 |
|  |  | More than 1 year but less than 5 years | | 3 |
|  |  | More than 5 years | | 4 |
|  | What are your highest medical qualifications? First ask if doctor or nurse, and then have separate questions on qualifications for each category | Doctor: | MBBcH | 1 |
|  |  |  | MBBcH with specialization  Specify: _______________________ | 2 |
|  |  |  | Other,  Please specify: _____________________ | 3 |
|  |  | Nurse: | BSc Nursing/M. Nursing | 4 |
|  |  |  | Diploma in nursing | 5 |
|  |  |  | Masters in nursing (e.g. M.soc) | 6 |
|  |  |  | Other,  Please specify: _____________________ | 7 |
|  | Type of facility  **OBSERVE. DO NOT ASK.** | Medical doctor in the public sector | | 1 |
|  |  | Medical doctor in the private sector | | 2 |
|  |  | Nurse in the public sector | | 3 |
|  |  | Nurse in the private sector | | 4 |
|  |  | District hospital | | 5 |
|  |  | Private hospital | | 6 |
|  |  | DAY hospital/clinic | | 7 |
|  |  | Others, please specify:  __________________________________________ | | 8 |
|  | What is the average monthly household income of your typical patients? | Low income (less than R1500/month) | | 1 |
|  |  | Low-middle income (R1500 – R5000/month) | | 2 |
|  |  | Middle income (R5000 – R10000/month) | | 3 |
|  |  | Middle-high income (R10 000 – R15 000/month) | | 4 |
|  |  | High income (more than 15 000/month) | | 5 |
|  |  | Don’t know | | 6 |
|  | On average, how many patients (with any disease or problem) do you see per day: | 0-2 | | 1 |
|  |  | 3-10 | | 2 |
|  |  | 11-20 | | 3 |
|  |  | 21-30 | | 4 |
|  |  | 31-50 | | 5 |
|  |  | 51-100 | | 6 |
|  |  | 101-200 | | 7 |
|  |  |  | |  |
|  | On average, how many patients with suspected tuberculosis do **YOU** see in a month? | 0-2 | | 1 |
|  |  | 3-10 | | 2 |
|  |  | 11-20 | | 3 |
|  |  | 21-30 | | 4 |
|  |  | 31-50 | | 5 |
|  |  | 51-100 | | 6 |
|  |  | 101-200 | | 7 |
|  |  | Other, please specify:  _________________________________________  _________________________________________ | | 8 |
|  | Do you treat TB patients yourself, or do you refer them to another center (e.g. public TB clinic or hospital)? | I treat patients | | 1 |
|  |  | Patients are referred another center | | 2 |
|  | What is the most common infectious disease you diagnose?  NB: multiple answers are allowed | Diarrheal disease | | 1 |
|  |  | Respiratory tract infection | | 2 |
|  |  | Sexually transmitted infections (other than HIV) | | 3 |
|  |  | HIV/AIDS | | 4 |
|  |  | Tuberculosis | | 5 |
|  |  | Hepatitis | | 6 |
|  |  | Other, please specify:  _______________________________________ | | 7 |
|  | What is your typical fee for an initial consultation? | Medical aid patients: | | 1 |
|  |  | R. __________________ | |  |
|  |  | Non-medical aid patients: | | 2 |
|  |  | R. __________________ | |  |
|  |  | No charge (zero Rand) | | 3 |
|  |  | Does not want to reveal | | 4 |

|  | Do you perform any of the following rapid tests to make quick management decisions in the same visit (i.e. while the patient waits)? How many, who interprets the result, and how much do your patients pay for the testing: | | | | | | | | | | | | | | | | | | | | | | |
| --- | --- | --- | --- | --- | --- | --- | --- | --- | --- | --- | --- | --- | --- | --- | --- | --- | --- | --- | --- | --- | --- | --- | --- |
|  | **Rapid test** | **Do you do this test in your clinic or practice?** | | | **If yes, on an average, how many of this test do you do in a month?** | **Time to get the test results? [record average number of min]** | **Who performs the rapid test?** | | | | **Who interprets or reads the results of the rapid test?** | | | **Do you make any treatment decisions on the basis of the rapid test results?** | | **How much do patients pay for each test? (ZAR) [if no charge, please write ZERO]** | **Do you keep a record of the test results?** | | **Does the patient receive post-test counseling?** | | | **Do patients usually wait on site for the rapid test results?** | |
|  |  | Yes | No | Not in clinic but lab attached to clinic |  |  | Provider | Support staff (e.g. nurse, compounder) | Attached lab | Provider | | Support staff (e.g. nurse, compounder) | Lab attached | Yes | No |  | Yes | No | Yes | No | Refer patient | Yes | No |
|  | 1. Pregnancy |  |  |  | tests/month | min |  |  |  |  | | | |  |  | ZAR |  |  |  |  |  |  |  |
|  | 1. Glucose (blood or urine sugar) |  |  |  | tests/month | min |  |  |  |  | | | |  |  | ZAR |  |  |  |  |  |  |  |
|  | 1. HIV |  |  |  | tests/month | min |  |  |  |  | | | |  |  | ZAR |  |  |  |  |  |  |  |
|  | 1. Malaria |  |  |  | tests/month | min |  |  |  |  | | | |  |  | ZAR |  |  |  |  |  |  |  |
|  | 1. GeneXpert for TB |  |  |  | tests/month | min |  |  |  |  | | | |  |  | ZAR |  |  |  |  |  |  |  |
|  | 1. Syphilis |  |  |  | tests/month | min |  |  |  |  | | | |  |  | ZAR |  |  |  |  |  |  |  |
|  | 1. Hepatitis   Specify A, B or C  _________________ |  |  |  | tests/month | min |  |  |  |  | | | |  |  | ZAR |  |  |  |  |  |  |  |
|  | 1. Influenza (flu) |  |  |  | tests/month | min |  |  |  |  | | | |  |  | ZAR |  |  |  |  |  |  |  |
|  | 1. Dengue |  |  |  | tests/month | min |  |  |  |  | | | |  |  | ZAR |  |  |  |  |  |  |  |
|  | 1. Typhoid |  |  |  | tests/month | min |  |  |  |  | | | |  |  | ZAR |  |  |  |  |  |  |  |
|  | 1. Streptococcal pharyngitis (throat) |  |  |  | tests/month | min |  |  |  |  | | | |  |  | ZAR |  |  |  |  |  |  |  |
|  | 1. Blood gas |  |  |  | tests/month | min |  |  |  |  | | | |  |  | ZAR |  |  |  |  |  |  |  |
|  | 1. Others (specify)   ______________ |  |  |  | tests/month | min |  |  |  |  | | | |  |  | ZAR |  |  |  |  |  |  |  |

| Q. No | Questions and Filters | Coding Categories | Codes |
| --- | --- | --- | --- |
|  | For the rapid tests you perform, what are the most important reasons for doing this in your practice or clinic, instead of sending your patients to an outside laboratory (e.g. NHLS or private lab service)?  Multiple answers are allowed  Do not read out the options | Convenience for patients | 1 |
|  |  | More affordable for patients | 2 |
|  |  | Results available immediately | 3 |
|  |  | To make a diagnosis and treatment decision within the same clinic visit | 4 |
|  |  | Other, please specify: | 5 |
|  |  | Not applicable | 6 |
|  | For the rapid tests you DO NOT perform, what are the most important reasons for **NOT** doing rapid testing in your practice?  Multiple answers are allowed  Do not read out the options | Too busy; do not have the time to do any testing | 1 |
|  |  | Lack of expertise to perform the rapid tests | 2 |
|  |  | Lack of capacity to perform the rapid tests | 3 |
|  |  | The available rapid tests are too expensive | 4 |
|  |  | Centralized lab services exist | 5 |
|  |  | Lack of quality assurance of POC testing results | 6 |
|  |  | Incentives for using external lab services | 7 |
|  |  | POC testing is not recommended by guidelines | 8 |
|  |  | Our clinic does not have an attached lab | 9 |
|  |  | Not profitable for clinic to conduct testing | 10 |
|  |  | Other, please specify: | 11 |

|  | The following questions are only about tuberculosis. If you send samples to an off site laboratory (e.g. NHLS or private labs) for TB testing: | | | | | | | | | | | | | | |
| --- | --- | --- | --- | --- | --- | --- | --- | --- | --- | --- | --- | --- | --- | --- | --- |
|  | Type of suspected TB | Not applicable because I have never seen a patient with this form of TB  If this box is checked, then skip question 17 and/or 18 appropriately | Do you diagnose or refer patients to another facility or clinic | | To which laboratory do you send the sample for testing? | | On average, how many patients or samples do you send for testing per month? | How long before you receive patient results? | Do you keep a record of all patients’ lab results? | | What is the average time between TB suspect being seen first in the clinic to starting of anti-TB treatment | Do you start TB treatment empirically, while you wait for the laboratory results? | | If you have to send samples, is there a well set-up specimen transport system? | |
|  |  |  | Diagnose | Refer patients | Public (NHLS) | Private (Pathcare, AMPTH, etc.) |  |  | Yes | No |  | Yes | No | Yes | No |
|  | Pulmonary TB |  |  |  |  |  | samples/month | days |  |  | Hours |  |  |  |  |
|  | MDR-TB |  |  |  |  |  | samples/month | days |  |  | Hours |  |  |  |  |
|  | XDR-TB |  |  |  |  |  | samples/month | days |  |  | Hours |  |  |  |  |
|  | Extra-pulmonary TB |  |  |  |  |  | samples/month | days |  |  | Hours |  |  |  |  |
|  | Other, please specify: |  |  |  |  |  | samples/month | days |  |  | Hours |  |  |  |  |

|  | What tests do you usually order for diagnosis of pulmonary TB (drug sensitive)? | | | | | | | | | | | | |
| --- | --- | --- | --- | --- | --- | --- | --- | --- | --- | --- | --- | --- | --- |
| **Test** | | **Is the test requested?** | | How important is this test in making a diagnosis of tuberculosis?  Please grade the importance of the TB diagnostic tests  *1 = not important 10 = extremely important* | | | | | | | | | |
| Chest X–ray | | Yes | No | 1 | 2 | 3 | 4 | 5 | 6 | 7 | 8 | 9 | 10 |
| Sputum smear examination | | Yes | No | 1 | 2 | 3 | 4 | 5 | 6 | 7 | 8 | 9 | 10 |
| Mantoux skin test (TST) | | Yes | No | 1 | 2 | 3 | 4 | 5 | 6 | 7 | 8 | 9 | 10 |
| Sputum culture | | Yes | No | 1 | 2 | 3 | 4 | 5 | 6 | 7 | 8 | 9 | 10 |
| PCR Eg. Hain MDR-TB (version 2.0) | | Yes | No | 1 | 2 | 3 | 4 | 5 | 6 | 7 | 8 | 9 | 10 |
| TB-Gold test (Quantiferon-TB Gold) | | Yes | No | 1 | 2 | 3 | 4 | 5 | 6 | 7 | 8 | 9 | 10 |
| GeneXpert | | Yes | No | 1 | 2 | 3 | 4 | 5 | 6 | 7 | 8 | 9 | 10 |
| Urine LAM test | | Yes | No | 1 | 2 | 3 | 4 | 5 | 6 | 7 | 8 | 9 | 10 |
| I do not order any TB tests – I refer patients to other centers or hospitals | | Yes | No | 1 | 2 | 3 | 4 | 5 | 6 | 7 | 8 | 9 | 10 |
| Serological or blood antibody tests (ELISA) | | Yes | No | 1 | 2 | 3 | 4 | 5 | 6 | 7 | 8 | 9 | 10 |

|  | What tests do you usually order for diagnosis of Multi-drug resistant TB (MDR-TB)? | | | | | | | | | | | | |
| --- | --- | --- | --- | --- | --- | --- | --- | --- | --- | --- | --- | --- | --- |
| **Test** | | **Is the test requested?** | | How important is this test in making a diagnosis of MDR-TB?  Please grade the importance of the TB diagnostic tests  *1 = not important 10 = extremely important* | | | | | | | | | |
| Chest X–ray | | Yes | No | 1 | 2 | 3 | 4 | 5 | 6 | 7 | 8 | 9 | 10 |
| Sputum smear examination | | Yes | No | 1 | 2 | 3 | 4 | 5 | 6 | 7 | 8 | 9 | 10 |
| Mantoux skin test (TST) | | Yes | No | 1 | 2 | 3 | 4 | 5 | 6 | 7 | 8 | 9 | 10 |
| Sputum culture | | Yes | No | 1 | 2 | 3 | 4 | 5 | 6 | 7 | 8 | 9 | 10 |
| Drug susceptibility testing (DST) | | Yes | No | 1 | 2 | 3 | 4 | 5 | 6 | 7 | 8 | 9 | 10 |
| PCR or polymerase chain reaction | | Yes | No | 1 | 2 | 3 | 4 | 5 | 6 | 7 | 8 | 9 | 10 |
| TB-Gold test (Quantiferon-TB Gold) | | Yes | No | 1 | 2 | 3 | 4 | 5 | 6 | 7 | 8 | 9 | 10 |
| I do not order TB tests for MDR-TB – I refer patients to other centers or hospitals | | Yes | No | 1 | 2 | 3 | 4 | 5 | 6 | 7 | 8 | 9 | 10 |
| Any other, please- specify:  _____________________ | | Yes | No | 1 | 2 | 3 | 4 | 5 | 6 | 7 | 8 | 9 | 10 |

|  | What tests do you usually order for diagnosis of Extra-pulmonary TB? | | | | | | | | | | | | | |
| --- | --- | --- | --- | --- | --- | --- | --- | --- | --- | --- | --- | --- | --- | --- |
| **Test** | | **Is the test requested?** | | **Please grade the importance of the TB diagnostic tests**  ***1 = not important 10 = extremely important*** | | | | | | | | | |  |
| Chest X–ray | | Yes | No | 1 | 2 | 3 | 4 | 5 | 6 | 7 | 8 | 9 | 10 |  |
| Smear examination of extrapulmonary specimens (e.g. fluids or biopsy from site of disease) | | Yes | No | 1 | 2 | 3 | 4 | 5 | 6 | 7 | 8 | 9 | 10 |  |
| Mantoux skin test (TST) | | Yes | No | 1 | 2 | 3 | 4 | 5 | 6 | 7 | 8 | 9 | 10 |  |
| Biopsy (tissue) from the site of disease | | Yes | No | 1 | 2 | 3 | 4 | 5 | 6 | 7 | 8 | 9 | 10 |  |
| TB-Gold test (Quantiferon-TB Gold) | | Yes | No | 1 | 2 | 3 | 4 | 5 | 6 | 7 | 8 | 9 | 10 |  |
| PCR assay (from site of disease) | | Yes | No | 1 | 2 | 3 | 4 | 5 | 6 | 7 | 8 | 9 | 10 |  |
| GeneXpert (from site of disease) | | Yes | No | 1 | 2 | 3 | 4 | 5 | 6 | 7 | 8 | 9 | 10 |  |
| TB-Gold test (Quantiferon-TB Gold) | | Yes | No | 1 | 2 | 3 | 4 | 5 | 6 | 7 | 8 | 9 | 10 |  |
| Culture of biopsy tissue or fluids from site of the disease | | Yes | No | 1 | 2 | 3 | 4 | 5 | 6 | 7 | 8 | 9 | 10 |  |
| I do not order TB tests – I refer patients to other centers or hospitals | | Yes | No | 1 | 2 | 3 | 4 | 5 | 6 | 7 | 8 | 9 | 10 |  |
| Any other, please specify:  ____________________________________ | | Yes | No | 1 | 2 | 3 | 4 | 5 | 6 | 7 | 8 | 9 | 10 |  |

| **Q. No** | **Questions and Filters** | **Coding Categories** | **Codes** |
| --- | --- | --- | --- |
|  | If you have to perform only **ONE TEST** for pulmonary TB, which one would you order for your patients with suspected pulmonary TB? | Chest X–ray | 1 |
|  |  | Clinical examination | 2 |
|  |  | Sputum smear examination | 3 |
|  |  | Mantoux skin test | 4 |
|  |  | Sputum culture | 5 |
|  |  | Drug susceptibility testing | 6 |
|  |  | PCR Eg. Line probe assay | 7 |
|  |  | GeneXpert | 8 |
|  |  | TB-Gold test (Quantiferon-TB Gold) | 9 |
|  |  | I do not order TB tests – I refer patients to other centers or hospitals | 10 |
|  |  | Serological or blood antibody tests (ELISA) | 11 |
|  |  | Any other, please specify:  ________________________________ | 12 |
|  | What is the reason for selecting the test (s) mentioned above?  **(Prompt and please keep asking by adding ‘and’ and record them till the person says no more. Multiple responses possible.)** | Affordable for my patient | 1 |
|  |  | Results are accurate | 2 |
|  |  | Quick time to results | 3 |
|  |  | Easily available in my setting | 4 |
|  |  | Recommended by national/ international guidelines | 5 |
|  |  | Incentives for ordering the test associated | 6 |
|  |  | Does not require sputum specimens | 7 |
|  |  | Helpful for childhood TB or extra-pulmonary TB | 8 |
|  |  | My patients prefer this test | 9 |
|  |  | Test is promoted by my local lab | 10 |
|  |  | I am not sure | 11 |
|  |  | Other, please specify:  __________________________________ | 12 |
|  | If you suspect tuberculosis in your patient (or have already diagnosed TB), do you routinely ask the patient to get tested for HIV? | Yes | 1 |
|  |  | No | 2 |
|  |  | Sometimes | 3 |

| **Q. No** | **Questions and Filters** | **Coding Categories** | **Codes** | **Codes for grading** | | | | | | | | | |
| --- | --- | --- | --- | --- | --- | --- | --- | --- | --- | --- | --- | --- | --- |
|  |  |  | **Criteria** | *Not important Extremely important* | | | | | | | | | |
|  | Imagine a test for TB diagnosis that could be done here in your primary care setting. What criteria should this point-of-care test have?  *Grade importance*  *1 = not important*  *10 = extremely important* | Rapid **(ask participant to specify in Q23)** | 1 | 1 | 2 | 3 | 4 | 5 | 6 | 7 | 8 | 9 | 10 |
|  |  | Cheap **(ask participant to specify in Q24)** | 2 | 1 | 2 | 3 | 4 | 5 | 6 | 7 | 8 | 9 | 10 |
|  |  | User friendly | 3 | 1 | 2 | 3 | 4 | 5 | 6 | 7 | 8 | 9 | 10 |
|  |  | Good specificity and sensitivity | 4 | 1 | 2 | 3 | 4 | 5 | 6 | 7 | 8 | 9 | 10 |
|  |  | Ease of sample acquisition (e.g. blood versus urine) | 5 | 1 | 2 | 3 | 4 | 5 | 6 | 7 | 8 | 9 | 10 |
|  |  | Reliable **(ask participant to specify in Q25)** | 6 | 1 | 2 | 3 | 4 | 5 | 6 | 7 | 8 | 9 | 10 |
|  |  | Requires little or no laboratory infrastructure | 7 | 1 | 2 | 3 | 4 | 5 | 6 | 7 | 8 | 9 | 10 |
|  |  | Low infection risk | 8 | 1 | 2 | 3 | 4 | 5 | 6 | 7 | 8 | 9 | 10 |
|  |  | Minimal calibration /maintenance of test is necessary | 9 | 1 | 2 | 3 | 4 | 5 | 6 | 7 | 8 | 9 | 10 |
|  |  | Should pick up both TB and drug-resistance at the same time | 10 | 1 | 2 | 3 | 4 | 5 | 6 | 7 | 8 | 9 | 10 |
|  |  | Should work well in HIV-infected patients | 11 | 1 | 2 | 3 | 4 | 5 | 6 | 7 | 8 | 9 | 10 |
|  |  | Other: _____________ | 12 | 1 | 2 | 3 | 4 | 5 | 6 | 7 | 8 | 9 | 10 |
|  |  | Other: _____________ | 13 | 1 | 2 | 3 | 4 | 5 | 6 | 7 | 8 | 9 | 10 |

|  | Specify the following definitions (ask immediately if mentioned under | What did you mean by “Rapid”?: *Record Verbatim* |
| --- | --- | --- |
|  |  |  |
|  |  |  |
|  |  |  |
|  |  |  |
|  | Specify the following definitions (ask immediately if mentioned under | What did you mean by “Cheap”?: *Record Verbatim* |
|  |  |  |
|  |  |  |
|  |  |  |
|  |  |  |
|  | Specify the following definitions (ask immediately if mentioned under | What did you mean by “Reliable”?: *Record Verbatim* |
|  |  |  |
|  |  |  |
|  |  |  |
|  |  |  |

|  | What are your major challenges and frustrations related to diagnosis of TB? Record Verbatim and **list** the most important reason first:  __________________________________________________________________________________________________________________________________________________________________________________________________________________________________________________________________________________________________________________________________________________________________________________________________________________________________________________________________________________________________________________________________________________________________________________________________________________________________________________________________________________________________________________________________________________________________ |
| --- | --- |

**The last section focuses on HIV**

| **Q. No** | **Questions and Filters** | **Coding Categories** | **Codes** |
| --- | --- | --- | --- |
|  | **In a month, how many HIV patients do you examine in your practice?** | Zero | 1 |
|  |  | 1-50 | 2 |
|  |  | 51-100 | 3 |
|  |  | 101-150 | 4 |
|  |  | 151 or more | 5 |
|  | **How many of these patients report having self tested for HIV in the past?** | Zero | 1 |
|  |  | 1-25 | 2 |
|  |  | 26-50 | 3 |
|  |  | 51-75 | 4 |
|  |  | 76 -100 | 5 |
|  |  | 101-or more | 6 |
|  | Are you aware that an oral (saliva) based test for HIV was recently approved by the US FDA for over the counter sale as a HIV self-test? | Yes | 1 |
|  |  | No | 2 |
|  | **Currently only 50% of South Africans test for HIV once in their lifetime. Will self-testing help reach more South Africans who do not wish to get tested for HIV in hospitals?** | Yes  Give a reason: | 1 |
|  |  | No  Give a reason: | 2 |
|  | Should Self tests for HIV be offered to South Africans? | Yes  Give a reason: | 1 |
|  |  | No  Give a reason: | 2 |
|  |  | No opinion | 3 |
|  | In the US, the self testing model is based on self tests sold over the pharmacies, coupled with a linked free 1-800-number to reach counselors Confidential testing, treatment and referrals are to be arranged with their assistance.  Do you think such a model will work in South Africa? | Yes  Why? | 1 |
|  |  | No  Why not? | 2 |
| **Q. No** | **Questions and Filters** | **Coding Categories** | **Codes** |
|  | In your opinion, how should we offer confidential linkages to counseling, and treatment to those South Africans that will self test themselves? | Face to face pharmacy counseling | 1 |
|  |  | Face to face clinic based counseling | 2 |
|  |  | Phone based counseling | 3 |
|  |  | Internet based counseling | 4 |
|  |  | No counseling | 5 |

|  | On a scale of 1 to 5.  1 being highly likely and 5 being least likely, what could be potential risks/negative effects associated with self-testing and how likely are those negative effects?” | Extreme emotional response in self test positive | 1 | 2 | 3 | 4 | 5 |
| --- | --- | --- | --- | --- | --- | --- | --- |
|  |  | Self harm in self test positive | 1 | 2 | 3 | 4 | 5 |
|  |  | Failure to initiate contact with counselors in self test negatives | 1 | 2 | 3 | 4 | 5 |
|  |  | Failure to initiate contact with counselors in self test positives. | 1 | 2 | 3 | 4 | 5 |
|  |  | Increase in risky behavior in self test negatives | 1 | 2 | 3 | 4 | 5 |
|  |  | Decrease in risky behavior in self test positives. | 1 | 2 | 3 | 4 | 5 |

**THANKS FOR YOUR VALUABLE TIME**
